# FlyVar: a database for genetic variation in *Drosophila melanogaster*

**DOI:** 10.1093/database/bav079

**Published:** 2015-08-18

**Authors:** Fei Wang, Lichun Jiang, Yong Chen, Nele A. Haelterman, Hugo J. Bellen, Rui Chen

**Affiliations:** ^1^Shanghai Key Lab of Intelligent Information Processing, School of Computer Science and Technology, Fudan University, Shanghai, China;; ^2^Human Genome Sequencing Center,; ^3^Department of Molecular and Human Genetics, and; ^4^Program in Developmental Biology, Baylor College of Medicine, Houston, TX, USA;; ^5^Howard Hughes Medical Institute

## Abstract

FlyVar is a publicly and freely available platform that addresses the increasing need of next generation sequencing data analysis in the *Drosophila* research community. It is composed of three parts. First, a database that contains 5.94 million DNA polymorphisms found in *Drosophila melanogaster* derived from whole genome shotgun sequencing of 612 genomes of *D*. *melanogaster*. In addition, a list of 1094 dispensable genes has been identified. Second, a graphical user interface (GUI) has been implemented to allow easy and flexible queries of the database. Third, a set of interactive online tools enables filtering and annotation of genomic sequences obtained from individual *D*. *melanogaster* strains to identify candidate mutations. FlyVar permits the analysis of next generation sequencing data without the need of extensive computational training or resources.

**Database URL**: www.iipl.fudan.edu.cn/FlyVar.

## INTRODUCTION

With the rapid progress of next generation sequencing (NGS) technology, analysis and especially interpretation of large amounts of sequencing data is complex. One of the most common applications of NGS technology is to identify causative mutations. Unfortunately, the vast majority of genomic variants in any individual are polymorphisms that do not affect gene function in an obvious fashion (1). One of the most effective ways to distinguish a damaging mutation from a polymorphism is by comparing affected and control genomes. In general, variations that show no enrichment in affected individuals, and/or are shared by a large number of controls, are likely to be benign polymorphisms and can often be excluded from further investigation (2, 3). The efficiency of this filtering step largely depends on the size of the control cohort. In humans, several large scale projects have been carried out to categorize variants of the human genome, such as the 1000 genomes project (4). Results obtained from these projects are essential resources for human genetics research. Similar NGS based methods have also been shown to be practical for mutation identification in model organisms, including *D. melanogaster* (5, 6). However, the key challenge is that the density of polymorphisms is high among fly strains. Recent studies of natural populations indicate the presence of more than 1 polymorphism per 500 base pairs in the fly genome, more than two times higher than that in the human genome (6–8). As a result, identification of a causative mutation among such a large number of polymorphisms is challenging. The best approach to solve this issue is to sequence parental strains from which a mutant is derived and use it as the control to identify variants that are unique to the mutant strain (9). However, this approach cannot be applied to the vast majority of the existing mutants that have been accumulated over the past 100 years as their parental strains do not exist anymore. Alternatively, similar to the human genetics field, a database containing a large number of variants observed from control individuals could be used to filter out polymorphisms. Existing databases usually contain a relatively small number of variants, ranging from several hundreds to tens of thousands (10–13). Recently dbSNP reports 5.2 million sequence variations for *D. melanogaster*, in which these variants could be queried. While, none of these databases offer variant filtering tools that remove out benign variants from raw sequence data to help narrow down mutation candidates.

To resolve this issue, we have constructed a database, FlyVar (www.iipl.fudan.edu.cn/FlyVar), by collecting data from both our own and public whole genome sequencing (WGS) projects. Currently, FlyVar contains 4.86 million single nucleotide polymorphisms (SNPs) and 1.08 million indels. A set of web based interactive tools has been developed to enable easy filtering of each variant to evaluate and prioritize variants. Through the GUI, users will be able to efficiently analyse individual fruit fly genome data without the requirement of extensive bioinformatics skills or computational resources.

## DATA SOURCE AND PROCESSING

Variants were collected from both our internal data and public data. Most of the internal data comes from the sequencing of a collection of X chromosome lethal mutants (9). In brief, EMS mutagenesis was performed and individual fly strains carrying lethal mutations on the X chromosome were established. Since the parental fly strains used for mutagenesis were isogenized for the X chromosome only, WGS of individual mutant strains allowed us to identify autosomal variants existing in the population of parental and balancer stocks. As a result, 0.92 million variants were identified. To further increase the size of the database, we have also deposited variants obtained from public sequencing data. Most of the public data are derived from the Drosophila Genome Research Project (DGRP) (14). For the DGRP, flies were collected at a food market in Raleigh, NC, USA and were used to establish many inbred strains, which were extensively phenotyped. WGS was performed for 205 inbred strains, from which 6.14 million variants were identified (15). As all these inbred strains are viable and free of apparent developmental defects, homozygous variants in these strains are likely to have minimal effects on development. Hence, a total of 4.7 million homozygous variants were identified and added into the FlyVar database. In addition, we have also included variants identified in four studies (7, 14, 16–18). All of these projects consisted of WGS of natural populations of *D*. *melanogaster*. From 49 WGS data sets, we were able to include an additional 0.32 million variants into the database. In total, 612 WGS data of *D. melanogaster* strains was collected into FlyVar, representing the most comprehensive data set.

## SUMMARY OF VARIANTS

A summary of variants by chromosome and their annotation is shown in Table 1 for SNPs and Table 2 for small insertion/deletions. To avoid potential mutations generated by EMS on X chromosome, only variants from the autosomes are included in our database for the X chromosome lethal screen project.

**Table 1.** Summary of SNPs in fly

SNPs/MNPs	Chr2L	Chr2R	Chr3L	Chr3R	ChrX	Chr4
No. sites overall	1 109 349	916 178	110 5112	1 079 279	638 526	13552
Variant density(per kb)	44.2539	43.4374	45.0892	38.7591	28.6962	10.4878
No. in Splicing	1013	1130	962	1130	403	20
No. in UTR3	26 354	28 092	27 875	30 862	17 759	469
No. in UTR5	20 774	21 906	22 029	23 413	11 741	286
No. in upstream^a^	74 083	70 417	73205	74 496	39 628	822
No. in downstream^b^	59 859	55128	60 261	59 652	34 243	744
No. in intergenic	351 864	238 941	349 703	302 888	206 443	3484
No. in intron	437 754	370 587	439 610	450 121	258 369	6271
No. in exon	137 657	129 977	131 467	136 717	69 940	1456
Synonymous	89 032	83 262	83 274	84 094	46 512	604
Nonsynonymous	48 058	46 159	47 688	52 017	23 224	842
Stop loss	65	75	57	83	26	0
Stop gain	502	481	448	523	178	10

^a^Upstream: variant overlaps 1-kb region upstream of transcription start site.

^b^Downstream: variant overlaps 1-kb region downstream of transcription end site.

**Table 2.** Summary of indels in fly

INDELs	Chr2L	Chr2R	Chr3L	Chr3R	ChrX	Chr4
No. sites overall	236 300	195 139	243 431	225 411	177 697	3927
Variant density (per kb)	10.2785	9.2518	8.7421	8.0950	7.9859	3.0391
No. in splicing	155	153	140	172	82	6
No. in UTR3	6711	7318	7371	8063	7135	134
No. in UTR5	4293	4432	4712	4613	3596	79
No. in upstream^a^	16 877	16 368	16 841	16 417	10 128	241
No. in downstream^b^	14 759	13 530	14 953	14 398	10 528	274
No. in intergetic	81 545	57 305	85 089	68 854	61 799	1137
No. in intron	108 098	92432	110 465	108 684	81 361	1991
No. in exon	3862	3601	3860	4210	3068	65
Synonymous	1353	1219	1103	1352	666	35
Nonsynonymous	2485	2351	2721	2832	2414	25
Stop loss	2	3	6	10	3	0
Stop gain	57	85	75	78	22	8

^a^Upstream: Variant overlaps 1-kb region upstream of transcription start site.

^b^Downstream: Variant overlaps 1-kb region downstream of transcription end site.

Both Tables 1 and 2 show that, the variant density, which is defined as the total number of variants per kb, is similar for the four arms of chromosome 2 and 3 at about 1 polymorphism per 20 base pairs. Variant density for the X chromosome is markedly lower. This is true even when we take the fact that variants on the X chromosome from the X chromosome lethal project are excluded. This is consistent with the idea that the X chromosome is under higher purify selection as males only have a single copy (19, 20).

**Figure 1.**
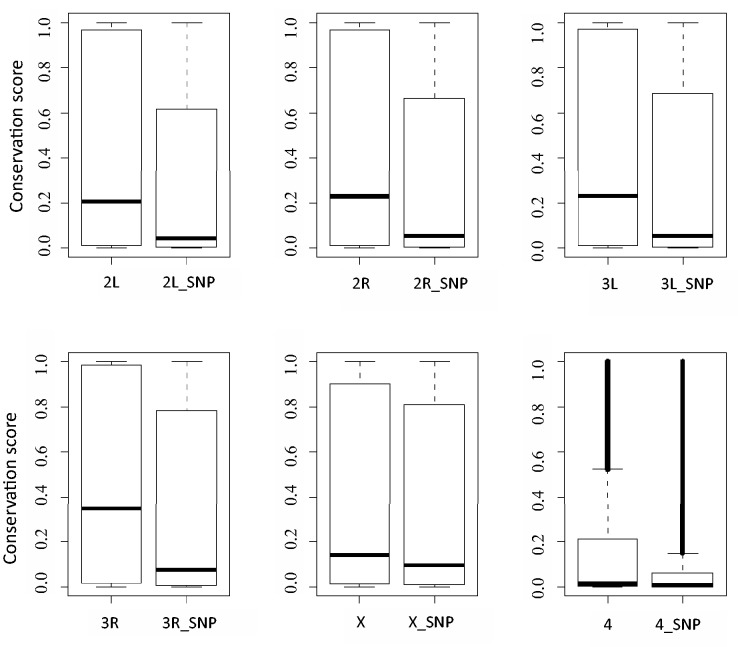
Comparison of evolutionary conservation scores between a whole chromosome and its polymorphism sites. In each plot, the left boxplot depicts conservation scores of a whole chromosome, such as ‘2L’ for chromosome 2L; the right boxplot displays conservation scores of which polymorphisms exists, for example ‘2L_SNP’ representing polymorphism sites of chromosome 2L.

As expected, the vast majority of the selected variants are likely to be benign polymorphisms for the following reasons. First, variants are enriched in noncoding region as about 70% of them are located in intergenic or intronic regions. Second, for variants that map to exons, 65% are synonymous, not affecting the amino acid sequence of the final protein product. Third, these variants tend to be less conserved bases. As shown in Figure 1, the average level of evolutionary conservation for the polymorphism bases is lower than that across the genome. It is worth noting that though many polymorphism sites are highly conserved, evolutionary conservation score alone is not sufficient to predict the functional importance of a given base.

Interestingly, we identified a total of 150 000 variants that corresponds to non-sense mutations, frame shifts and splice site mutations. These represent potential loss of function (LOF) mutations. These variants may affect genes that do not play an essential role in viability or cause an obvious phenotype (21). Second, a detrimental variant may be compensated for by a different variant, present elsewhere in the genome. Third, it is possible that a variant only affects a subset of transcript, isoforms or functional domains of a gene. This is particularly likely when a variant is located within alternatively spliced exons. Finally, nonsense mutations towards the end of a protein coding region may lead to a minor truncation of the protein with subtle functional consequences (22). To exclude the last two possibilities, we applied stringent criteria by which only LOF mutations that fall within constitutive exons and not within the last coding exons are considered. As a result, a total of 1094 genes that have clear LOF mutations when in a homozygous state in at least one strain is identified and these genes are considered dispensable for obvious developmental defects. When applied, this dispensable gene list allows exclusion of variants from these genes during the filtering process, further improving the selectivity in classifying deleterious mutations. We noticed that some dispensable genes are known lethal genes. Therefore, we provided an option for users whether to filter out variants within dispensable gene regions.

## PLATFORM FUNCTIONS

To facilitate the usage of the database by individual researcher, we have implemented several web based functionalities to the database, allowing querying, annotating and filtering.

**Figure 2.**
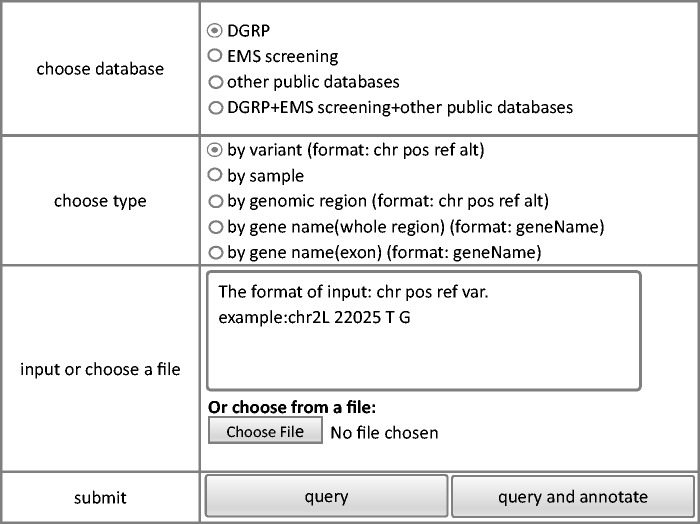
Steps of query model of FlyVar database.

The criteria based query module allows extraction of variance information from the database. The interface of the query module is shown in Figure 2. To make it easy for all users, we keep the interface of FlyVar very simple. Data can be queried based on fly strain ID, genomic coordinate, a given genomic interval, and gene ID. When users have chosen way of querying, corresponding input format and an example could be shown in the right input position of webpage. For example, as shown in Figure 2, the querying way was chosen as ‘by variant’, corresponding input format and an example ‘The format of input:chr pos ref var. example:chr2L 22025 T G’ is presented immediately in the box of‘input or choose a file’part. The query output includes chromosome, genomic coordinate, reference sequence, and variant sequence. In addition, the ID of the fly strain(s) in which the variant was identified is included. We also designed a query by sample function, in case the specific strain ID in a dataset is meaningful. Furthermore, to facilitate user access to the DGRP collection, all variants from individual DGRP strains can be obtained by querying the corresponding strain ID. Finally, variant frequency in the DGRP collection is also provided with links to the corresponding strains at the FlyBase website (23).

**Figure 3.**
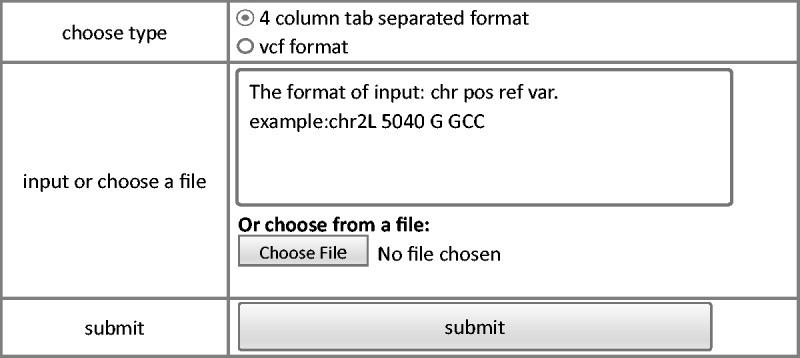
Steps of annotation model of FlyVar database.

The annotation module provides a user-friendly interface to annotate variants input by the users. Currently, Drosophila melanogaster Release 5.1 reference (23, 24) and an annotation tool ANNOVAR (25) are used to perform functional annotation. Webpage of annotation module is shown in Figure 3. Input with standard vcf format or user defined tab-separated plain text are both accepted. For the custom plain text file, only essential information to define a variant, including chromosome, coordinate, reference base, and variant base are required. This module will generate two output files: the first file contains annotation for all the variants while the second file contains annotation for only variants that affect protein coding.

**Figure 4.**
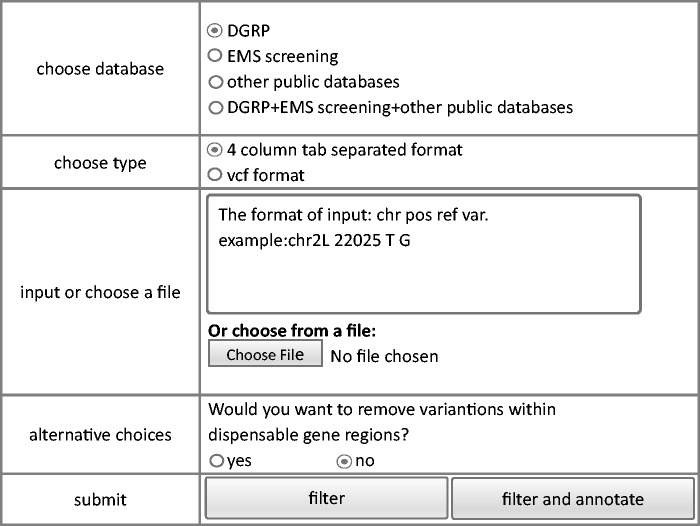
Steps of filtering model of FlyVar database.

We also developed a module that permits filtering of raw sequence data to eliminate potentially benign variants. The module removes homozygous polymorphisms stored in the database resulting in a narrower list of candidate mutations. To avoid potential deleterious mutations, only homozygous variants were considered benign and filtered out. Same style of filtering interface as query module and annotation module is shown in Figure 4. Similar to the annotation module, both vcf and user defined tab delimited files can be used as input. The output file has the same format as the input file and only contains variants that are not observed in the database.

Since some dispensable genes are known lethal genes, an option, whether variants in dispensable genes are filtered out or not, is provided. Based on the annotation file of collected LOF mutations which have been stored in our database and could be downloaded from ‘dispensable gene’ webpage, users could make their own decision about how to utilize dispensable genes. If dispensable gene regions were included into filtering process, the rest of variants after filtering would be separated into two parts, variants not in dispensable gene regions and variants within dispensable gene regions. Input variants within dispensable genes could also be annotated to ease decision making.

Considering that the number of variants that need to be processed can be large, we provide support for batch file input processing as well as a standard interactive interface. This interactive input feature allows processing of up to 200 000 variants at once. For a larger number of variants, a batch file containing the variants can be uploaded and results will be sent back to users through email.

Besides querying or annotating or filtering online, users also could download the whole database into their local disk.

Data downloading module organizes data according to its project source to ease data selection.

By now, FlyVar is a publicly and freely available for all potential users including profit and non-profit organizations. After logging in, users could give comments or suggestion.

## DISCUSSION

Here, we present a set of integrative web-based tools that allow individual research labs to process NGS data directly. This is the first of its kind that specifically addresses an increasing need for processing NGS data within the *Drosophila* research community. The FlyVar database contains data derived from the latest public WGS data. As a result, the number of variants collected in our database is several orders of magnitude larger than existing databases, greatly enhancing its utility. Upon filtering, the number of remaining variants will be dramatically reduced, resulting in a smaller, more accurate list of candidate mutations. The chrX mutation sequencing project delivered proof-of-principle of the usefulness of this database (9). Finally, to allow easy usage and access of the database, a set of interactive web based tools have been implemented that enable straightforward data processing without the requirement for programming or special computational resources.

There are several aspects that we are currently improving. First, copy number variations will be included in the near future. Second, we will further improve our annotation of variants. For example, annotation from the modENCODE (26) data can be included to annotate the potential effects a variant might have on a gene regulatory element. Third, as it is important to keep the database growing, we are developing tools that allow the community to easily contribute their private data. Finally, we plan to adopt cloud computing to the database and tools to make big data sets more accessible for the researchers.
